# Multi-omics profiling suggests deep brain stimulation-associated expansion of CLIC3+ NK cells attenuates neuroinflammation in Parkinson's disease

**DOI:** 10.1016/j.bbrep.2026.102861

**Published:** 2026-11-03

**Authors:** Shushan Zhang, Zhanghong Luo, Yishuai Tan, Bo Cheng, Wei Xiao, Guohui Jiang, Tao Zhu

**Affiliations:** a Department of Neurology, Affiliated Hospital of North Sichuan Medical College, Nanchong, Sichuan, China; b State Key Laboratory of Molecular Oncology & Department of Pathology, National Cancer Center/National Clinical Research Center for Cancer/Cancer Hospital, Chinese Academy of Medical Sciences and Peking Union Medical College, Beijing, 100021, China; c West China School of Pharmacy, Sichuan University, Chengdu, 610041, Sichuan, China; d School of Public Health, North Sichuan Medical College, Nanchong, Sichuan, China

## Abstract

Deep brain stimulation (DBS) alleviates Parkinson's disease (PD) symptoms, but its immunomodulatory mechanisms remain elusive. Through integrating microarray (GSE23676), single cell/nucleus RNA-seq (GSE184950, GSE202210, GSE223138, GSE243639; after quality control (mitochondrial gene content <25%, gene number >200) 616,076 high-quality cells were included), and GWAS data, we identified CLIC3 as a DBS-associated, potential protective implications in PD. CLIC3 was selectively up-regulated in a distinct natural killer cell subset (NK_CLIC3), which secreted TGFB1 to restrain neuroinflammation. CellChat and immunohistochemistry revealed that NK_CLIC3 cells communicated with PD-relevant neuronal populations: excitatory (SLC18A2+), inhibitory (SLC18A2+), oligodendrocyte (SLC18A2+), and astrocytic (SLC18A2+) cells via THY1–ADGRE5 and GZMA–PARD3 axes. Immune infiltration analysis (GSE114517; n = 75) suggested NK infiltration and deficient M2 macrophage recruitment in PD brains, underscoring an inadequate anti-inflammatory response. We further isolated CLIC3-positive NK cells (from a healthy donor) by fluorescence-activated cell sorting (FACS) and confirmed their ability to degrade α-synuclein, the pathological protein in Parkinson's disease, supporting the potential NK(CLIC3+) cells' protective function in PD. Our findings suggest DBS may expand NK_CLIC3 cells to suppress neuroinflammation through TGFB1 mediated immune–neuronal crosstalk, positioning CLIC3 as a prospective therapeutic target for PD.

## Highlights

• CLIC3 may serve as a DBS-induced PD protective factor.
• Immune infiltration shows deficient M2 macrophages in PD.
• SLC18A2^+^ neural subsets exhibit reduced proportions in PD.

## 1 Introduction

Parkinson's disease (PD) is the second most prevalent neurodegenerative disorder, characterized by progressive loss of dopaminergic neurons in the substantia nigra pars compacta and accumulation of α-synuclein aggregates [1], [2]. Despite advances in symptomatic management, disease-modifying therapies remain elusive [3], [4]. Deep Brain Stimulation (DBS) surgery is one of the most advanced neurosurgical methods for the treatment of Parkinson's disease [5]. It is an important treatment for patients with advanced Parkinson's disease. Advances in engineering and imaging, as well as an improved understanding of brain disease, promise to reshape the way DBS is viewed and delivered to patients [6]. Predicting the optimal stimulation contact based on local field potentials recordings is feasible and can improve DBS programming efficiency in PD [7]. However, the biological underpinnings of DBS, especially its effects on neuroinflammation and immune cell dynamics remain poorly understood.

Emerging evidence implicates immune dysregulation as a critical driver of PD pathogenesis [8]. Genome-wide association studies (GWAS) have linked Parkinson's disease to genes involved in immune responses, with genetic variation at HLA-DRB5 being associated with PD [9]. Additionally, single-cell RNA sequencing (scRNA-seq) analyses reveal profound alterations in brain-resident and peripheral immune cell populations in PD, including expansion of pro-inflammatory microglia and dysfunctional T cells [10]. Notably, natural killer (NK) cells which traditionally recognized for anti-tumor and antiviral functions have recently been implicated in neuroprotection via modulation of neuroinflammation [11].

Recent studies have identified CLIC3 as a core immune-related hub gene in schizophrenia [12]. Furthermore, the expression of CLIC3 in plasma and specific brain regions was significantly decreased in a rat model of schizophrenia, which was reversed by risperidone treatment, suggesting that CLIC3 may serve as a potential diagnostic biomarker and therapeutic target [12]. Our preliminary microarray data analysis of DBS-treated PD patients revealed significant upregulation of CLIC3 in peripheral blood, prompting investigation into its functional relevance. Then, we found CLIC3 was mainly expressed in natural killer (NK) cells. Here, we hypothesized that DBS is associated with CLIC3+ NK cell expansion, which subsequently attenuates neuroinflammation through paracrine signaling and cellular interaction. To test this, we integrated multi-omics datasets including DBS-associated transcriptomes, PD GWAS, and scRNA-seq/snRNA-seq from brain tissues and peripheral blood. This analysis uncovered a natural killer cell subset marked by CLIC3 (NK_CLIC3) and suggests a potential role of NK_CLIC3 in DBS-related effects. Cellular communication mapping further revealed that NK_CLIC3 engages PD-relevant neuronal subtypes namely SLC18A2^+^ excitatory neurons, inhibitory neurons, oligodendrocytes, and astrocytes via ligand–receptor pairs THY1–ADGRE5 and GZMA–PARD3. These findings provide mechanistic insights into DBS-associated neuroprotection and implicate CLIC3+ NK cells as a potentially tractable therapeutic target for PD.

## 2 Methods

1 Data acquisition

All brain tissue samples used in this study were post-mortem specimens obtained from public GEO repositories. GSE184950(23 PD and 11 control samples) [13] post-mortem substantia nigra (SN) tissues were sourced from NIH Neurobiobank (www.neurobiobank.nih.gov) and provided by the Brain Endowment Bank at the Miller School of Medicine. GSE202210 (6 PD patients and 6 control samples) [14] post-mortem prefrontal cortex tissues from PD patients were obtained from the brain tissue biorepository at NKI/NYUGSOM. GSE243639 [15] single-nucleus RNA-seq data were generated from SNpc of 29 post-mortem human brains (15 sporadic PD and 14 control samples) acquired from the Oregon Brain Bank. GSE114517 substantia nigra tissues were post-mortem samples from PD and non-PD donors provided by the Netherlands Brain Bank. The blood-derived datasets were as follows: GSE23676 (microarray data, 7 PD patients) [16] contains blood samples from patients receiving dopamine replacement therapy (DRT), who were examined pre-DBS and post-DBS under off-stimulation status with decreased DRT doses after DBS. GSE223138 (4 PD patients and 2 control samples) [17] includes PBMCs isolated from six participants for scRNA-seq to characterize cell subsets and differential gene expression. Integrative analysis was performed on scRNA/snRNA-seq data from a total of 81 samples, covering both brain tissue and peripheral blood specimens. After quality control (excluding cells with >25% mitochondrial reads or <200 detected genes) 616,076 cells remained. Batch effects were subsequently removed using the R package Harmony [18].

2 Microarray gene expression profiling data and scRNA/snRNA-seq data analysis

For microarray gene expression profiling data, differential gene expression analysis was performed using the limma package in R. Genes were considered differentially expressed genes (DEGs) if they met the criteria of adjusted P value < 0.05 (DEGs were screened based on Benjamini–Hochberg adjusted *P* values) and absolute log2 fold change > 1. The pheatmap R package was utilized to generate heatmaps for visualizing these DEGs. For single-cell RNA sequencing data analysis, the Seurat and stringr R packages were employed for data preprocessing and import. Furthermore, the harmony R package was used to eliminate batch effects between samples. The SCP R package was applied for visualizing cell dimensionality reduction, clustering, and gene expression levels. The FindAllMarkers function was used to identify representative genes among different cell subpopulations, which were subsequently subjected to enrichment analysis. Functional enrichment of DEGs (identifies differentially expressed genes between two groups of cells using a Wilcoxon Rank Sum test in scRNA/snRNA data) was performed using the clusterProfiler [19] package. Gene Ontology (GO) terms, including biological process, cellular component, and molecular function, were analyzed. Briefly, one-sided version of Fisher's exact test (over-representation test) was performed to identify enriched GO categories. P values in GO enrichment analysis were adjusted using Benjamini-Hochberg method.

3 Mendelian Randomization analysis

Summary-level data for exposure and outcome traits were obtained from public genome-wide association studies (GWAS) repositories (IEU OpenGWAS). Instrumental variables (IVs) were single nucleotide polymorphisms (SNPs) strongly associated with the exposure (P < 5 × 10^−8^) and in low linkage disequilibrium (LD, r^2^ < 0.001, window size = 10,000 kb). Using eQTL data for CLIC3 (exposure; ID: eqtl-a-ENSG00000169583; n = 31,684; 18,471 SNPs) as the genetic instrument, we performed two-sample Mendelian randomization against Parkinson's disease (outcome) represented by three independent GWAS: ieu-a-812 [20] (n = 5,691; 453,218 SNPs), finn-b-PD_DEMENTIA (n = 216,895; 16,380,459 SNPs), and finn-b-PD_DEMENTIA_EXMORE (n = 111,888; 16,379,396 SNPs). SNPs associated with the outcome or confounders were excluded. Two-sample MR was performed using the TwoSampleMR package. The inverse-variance weighted (IVW), weighted median, weighted mode, MR-Egger regression and simple mode methods were used to estimate causal effects.

4 Pseudotime analysis

Trajectory inference was performed using the Monocle3 [21] package to reconstruct pseudotime trajectories for inhibitory neurons and astrocytes. The Seurat object was used as input, from which the counts matrix, cell metadata, and gene annotations were extracted to construct a Monocle3 object. The root region on the UMAP projection was manually defined (with coordinates restricted to umap_1 ∈ [−5, 4.9] and umap_2 ∈ [−1, 0.9]) to identify root cells. Cells in this region exhibit high expression levels of 'TBR1’, 'DCX’, 'NEUROD1’, and 'TUBB3’, confirming their identity as neural progenitor cells. Subsequent pseudotime ordering was completed using the order_cells function, and trajectories were visualized with pseudotime-based coloring. To maintain consistency with Seurat integration results, the UMAP coordinates generated by Seurat were directly adopted for visualization.

5 Cell-Cell Communication analysis

A CellChat [22] object was constructed using the createCellChat function. Subsequently, identifyOverExpressedGenes and identifyOverExpressedInteractions were sequentially executed to identify overexpressed genes and potential intercellular interactions. The computeCommunProb function was used to calculate communication probabilities between each pair of cell clusters, followed by filterCommunication (min.cells = 10) to remove noise signals from low-abundance cell groups. Pathway-level communication networks were summarized using computeCommun ProbPathway and aggregateNet, generating two metrics: count and weight. Circular network plots (e.g., for interactions between NK cells and neuronal subpopulations) and bubble plots illustrating specified source-target relationships were visualized using netVisual_circle and netVisual_bubble, respectively.

6 Immunohistochemistry (IHC)

Expression levels of THY1, PARD3, and SLC18A2 in brain tissue were analyzed using The Human Protein Atlas database. For THY1, antibodies CAB068243 and CAB068244 were employed; for PARD3, antibody HPA030443; and for SLC18A2, antibody HPA073224.

7 Immune infiltration analysis

The gene expression dataset used for immune cell infiltration analysis is GSE114517 [23], which includes bulk RNA sequencing data from 75 brain tissue samples. Immune cell infiltration proportion estimation for the GSE114517 dataset was performed using the CIBERSORT R package. Algorithm cibersort deconvolves bulk RNA-seq data using a pre-validated signature matrix of immune cell marker genes, thereby quantitatively inferring the relative infiltration proportions of 22 immune cell subsets in each sample. All statistical analyses and visualizations were conducted using R software (version 4.2.0) with the following key R packages: dplyr, tibble, tidyr, ggplot2, ggthemes, ggpubr and pheatmap.

8. Flow Cytometric

Peripheral blood sample was obtained from one healthy volunteer, and the related research protocol was approved by the Ethics Committee of North Sichuan Medical College. For fluorescence-activated cell sorting of CLIC3-positive NK cells, 15 mL of Ficoll reagent (Cat: 17144002) was first added into a 50 mL centrifuge tube, which was then tilted at 30°, and 35 mL of diluted peripheral blood was gently layered onto the surface of Ficoll along the tube wall to prevent intermixing, followed by centrifugation at 400×g for 30–40 min at room temperature (20 °C). After carefully taking out the centrifuge tube, the buffy coat layer between the Ficoll solution and plasma, which represented peripheral blood mononuclear cells (PBMCs), was carefully aspirated and transferred into a new 50 mL centrifuge tube, and buffy coats from multiple tubes could be pooled if necessary. Subsequently, the collected cells were resuspended in 45 mL PBS and centrifuged at 300×g for 10 min at 20 °C, and this washing procedure was repeated twice before discarding the supernatant. The PBMCs were then resuspended in an appropriate buffer and counted, followed by incubation with labeled antibodies at 4 °C for 30 min. After being washed with PBS twice, the cell suspension was subjected to flow cytometric sorting, in which CD56^+^CD3^−^ NK cells were first collected, and the CLIC3-positive cell population was further isolated from the NK cell fraction to obtain the target NK-CLIC3 cells.

9 Western Blot

For protein extraction, 10^6^ cells were taken and lysed with 500 μL RIPA lysis buffer, to which 5 μL PMSF was added at a ratio of PMSF:RIPA = 1:100; steel beads were added, and the cells were homogenized on ice for 3–4 min each time to achieve sufficient lysis, then kept on ice for subsequent protein quantification or stored at −80 °C (during this process, 5 μL PMSF was added every 1 h). For protein quantification, the prepared standard BSA protein was diluted with sterile ddH_2_O to a concentration of 1 mg/mL; the 1 mg/mL standard sample was used to draw a standard curve, and the other standard samples were used for calibration; in a 96-well plate, 0, 1, 2, 4, 8, and 12 μL of the 1 mg/mL standard sample were added to the wells in sequence with 1 replicate well for each, followed by the sequential addition of 2 μL of standard samples and protein lysates, also with 1 replicate well for each; sample lysate and loading buffer were added to a new EP tube, mixed well, boiled at 95–100 °C for 10 min, centrifuged for 5 min after cooling, and 20–40 μg of total protein was loaded for WB analysis. The primary antibodies used for WB were as follows: β-actin (42 KDa) at a dilution of 1:100,000 (ABclonal, Cat: AC028) and α-syn (15–19 KDa) at a dilution of 1:1000 (Abcam, Cat: ab138501).

## 3 Results

1 CLIC3 is upregulated in peripheral blood after deep brain stimulation (DBS) treatment and may represent a factor with potential protective in Parkinson's disease (PD).

We performed differential gene analysis on microarray gene expression profiling data from PD patients before and after DBS treatment, as well as from normal control subjects (Fig. 1A–F). The results showed that CLIC3 was increased more than two-fold in the PDS3 group compared with the other three groups (HC, PDS1, and PDS2), respectively (Fig. 1C and E&F). All differential genes are presented in Supplementary Tables S1 and S2. Subsequently, we conducted a Mendelian randomization analysis with CLIC3 as the exposure factor and PD as the outcome, which suggests an association whereby CLIC3 may confer potential protective effects in PD (Fig. 1G–I). Additionally, the β values of the five analytical methods for each outcome were all less than 0, indicating that the negative correlation between CLIC3 and PD suggests that CLIC3 is a protective factor; among them, the p value obtained by the Weighted median method with finn-b-PD_DEMENTIA as the outcome was 0.02, and the p value obtained by the Weighted median method with finn-b-PD_DEMENTIA_EXMORE as the outcome was 0.03, both of which were statistically significant (Supplementary Table 3). To explore the specific cellular and biological mechanisms of CLIC3 in PD patients, integrated single-cell/nucleus sequencing data from 81 samples was obtained for further investigation. The samples included data from both peripheral blood and brain tissues, and all cells were clustered into 9 distinct cell subpopulations (Fig. 1J–L). These cell subtypes were characterized by the high expression of corresponding marker genes: Microglia (C3, LRMDA, DOCK8), Oligodendrocytes (MBP, PLP1, ST18), T/NK cells (CD3D, NKG7, GNLY), Excitatory neurons (CAMK2A, CBLN2, LDB2), Endothelial cells (CLDN5, ABCB1, EBF1), Inhibitory neurons (GAD1, LHFPL3, PCDH15), Astrocytes (AQP4, ADGRV1, GPC5, RYR3), Myeloid cells (CD68, S100A8, S100A9), and B cells (MS4A1, CD79A, CD19) (Fig. 1M). Analysis of CLIC3 expression patterns showed that CLIC3 is mainly expressed in the T/NK cell subpopulation (Fig. 1N). The average expression levels of CLIC3 in T/NK cell subsets were 1.29 (HC) and 1.38 (PD); the average levels in other cell subsets were all less than 0.1, and the second highest cell subset was B cell subset with 0.07 (HC) and 0.08 (PD). These results suggest that CLIC3 is mainly expressed in T/NK cell subsets and is slightly increased in PD patients (Supplementary Table 4). Integrative multi-omics analysis indicated that DBS treatment-associated upregulation of CLIC3 in peripheral blood may exert a positive modulatory effect relevant to PD. Furthermore, this upregulation may enhance the quantity or activity of T/NK cells, thereby further contributing to the effects relevant to PD management.

2 Single-cell integration analysis suggested that CLIC3 is predominantly expressed in natural killer (NK) cells and identification of an NK_CLIC3 Subset with Enhanced Immune-Activating Capacity

**Fig. 1:**
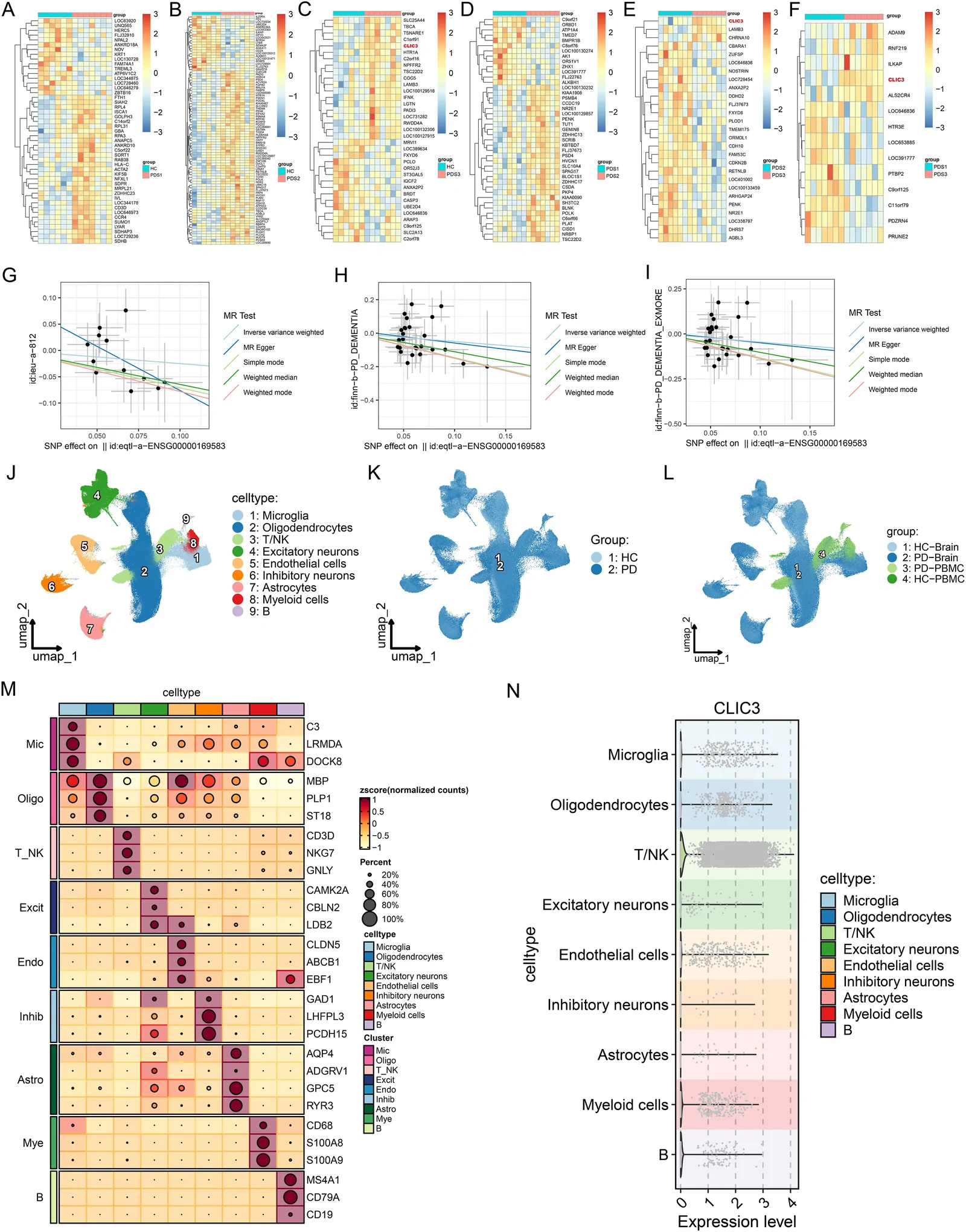
Transcriptomic and genetic analyses reveal CLIC3 as a protective factor in parkinson's disease (PD) and its enrichment in NK cells. (A–F), Heatmaps illustrating the expression patterns of differentially expressed genes between PD (before and after deep brain stimulation (DBS) therapy) and healthy control (HC) samples. Rows represent genes; columns represent samples, colored by normalized expression levels. HC indicates healthy controls, and PD indicates Parkinson's disease. S1 (pre-treatment), S2 (post STN-DBS), and S3 (upon off stimulation) represent three time points for PD patients, denoted as PDS1, PDS2, and PDS3, respectively. Each figure displays differentially expressed genes between the two indicated groups, with thresholds set at |log_2_FC| > 1 and adjusted P value < 1. (G–I), Mendelian randomization (MR) analyses depicting the causal relationship between CLIC3 and PD. Scatter plots show the effect of CLIC3 (x-axis) on PD (y-axis), with weighted regression lines (inverse variance weighted, MR Egger, Simple mode, weighted median and weighted mode) and indicating CLIC3 as a protective factor. In the dot-line plots, all diagonal lines slope downward, indicating that the β-values are less than 0, which demonstrates a negative correlation between the two factors. (J–L) UMAP visualizations of cell populations colored by cell type (J), disease group (K: HC vs. PD), and tissue origin (L: brain vs. peripheral blood mononuclear cells (PBMC)). (M) Dot plot of marker genes for major cell types, showing normalized expression (color) and cell-type-specific percentage (dot size). (N) Violin plot of CLIC3 expression across cell types, highlighting enrichment in T/NK cells.

After subclustering T cells and NK cells, they were divided into seven cell subpopulations: T_Naïve, T_Regulatory, T_Memory, T_Effector, NK_CLIC3, NK_KLRC1, and NK_KLRC2 (Fig. 2A and B). The overall proportion of NK cells in the T/NK cell population was lower in Parkinson's disease (PD) patients compared to normal control samples (Fig. 2C). Among these, the NK_CLIC3 subpopulation showed high expression of the genes NKG7, GNLY, FGFBP2, and CLIC3 (Fig. 2D). Additionally, CLIC3 was expressed in both normal controls and PD patients (Fig. 2E and F). Among them, the CLIC3 level in the NK_CLIC3 cell subset was the highest among all cell subsets, with average expression levels of 4.74 (HC) and 5.66 (PD); the expression levels of other NK cell subsets were all lower than 3.5, and the expression levels of T cell subsets were all lower than 1, which suggests that CLIC3 is mainly expressed in NK cells and shows the highest expression in the NK_CLIC3 subset (Supplementary Table 4). Enrichment analysis of the three NK cell subpopulations suggests that cytokine pathways and cell activation in immune-related pathways such as T/B cell receptor signaling pathway and cell activation involved in immune response were most strongly activated in the NK_CLIC3 subtype compared to the other two NK cell subpopulations (Fig. 2G). Through subpopulation refinement, we found that CLIC3 is primarily concentrated in the NK_CLIC3 subpopulation, which exhibits stronger activation of cytokine and immune response pathways. This result suggests that the NK_CLIC3 subpopulation may have a potential role relevant to DBS effects in PD patients.

3 Identification of PD-Associated Excitatory Neuronal Subpopulations through Sub-clustering Analysis

**Fig. 2:**
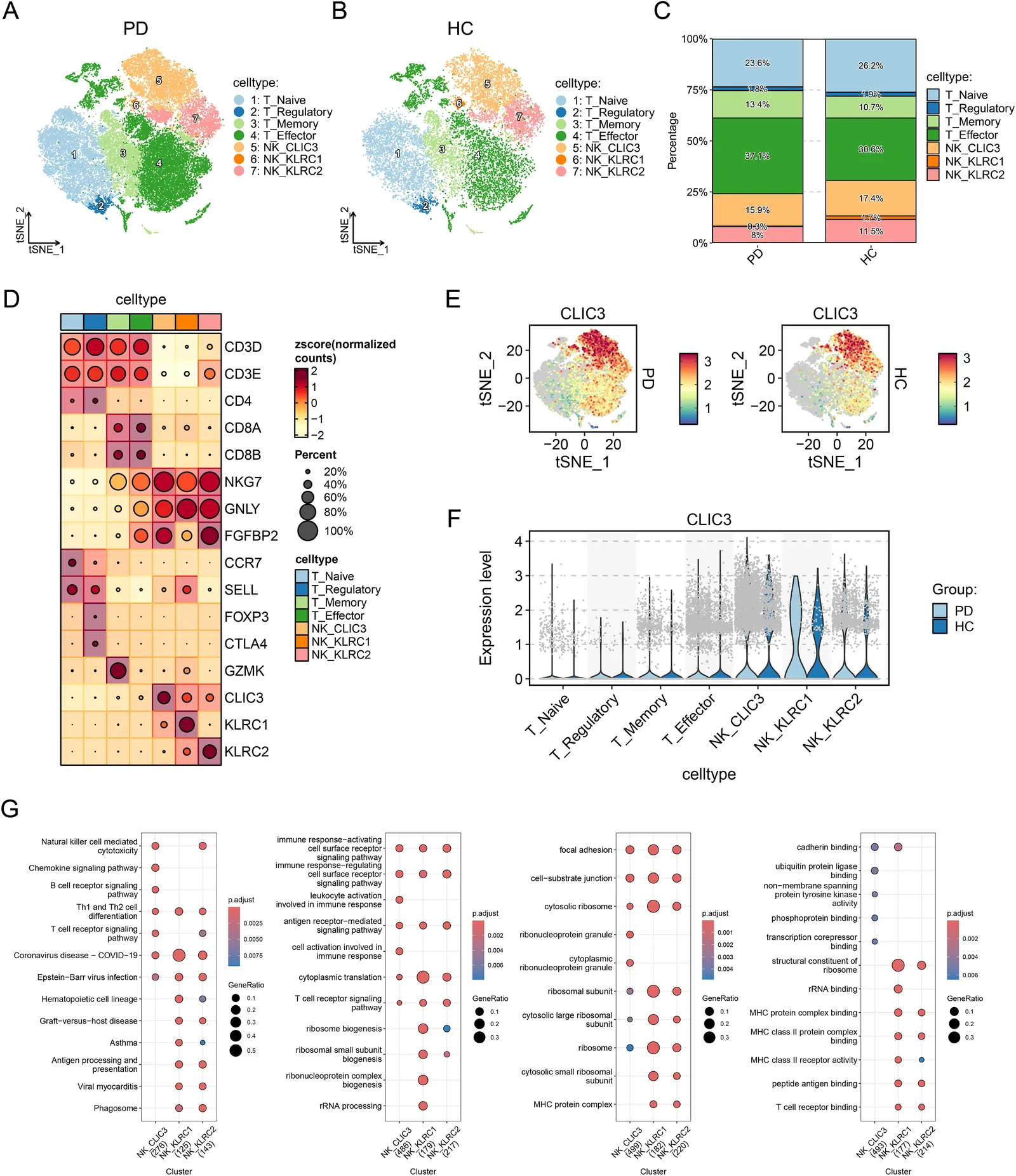
Single-cell profiling of T/NK cell subsets reveals CLIC3+ NK Cells as a distinct subpopulation in PD. (A–B) UMAP visualizations of T/NK cell subpopulations in PD (A) and HC (B) samples, colored by cell type. (C) Stacked bar plots showing the percentage distribution of T/NK cell subsets in PD and HC. (D) Dot plot of marker genes for T/NK cell subpopulations, with normalized expression (color) and cell-type-specific percentage (dot size). (E) UMAPs of CLIC3 expression in T/NK cells from PD (left) and HC (right) samples, colored by expression level. (F) Violin plots comparing CLIC3 expression across T/NK cell subpopulations in PD and HC. (G) Bubble plots of enriched pathways in CLIC3+ NK cells, showing pathway enrichment (y-axis), and gene ratio (dot size). Pathways are grouped by functional categories.

We extracted excitatory neuronal populations for sub-clustering analysis, which identified a total of 12 distinct cell subpopulations (Fig. 3A and B). Among these, the proportion of excitatory neurons (F3+) was higher in the Parkinson's disease (PD) patient group, while the proportion of the excitatory neurons (SLC18A2+) subpopulation was lower in PD patients compared to controls (Fig. 3C). Scoring of cell subpopulations based on PD-related genes (PARK7, LRRK2, etc.) revealed that two excitatory neuron subpopulations—excitatory neurons (SLC18A2+) and excitatory neurons (HTR2C+)—were particularly closely associated with PD (Fig. 3D and E). Differential gene analysis of excitatory neurons (SLC18A2+) between PD patients and healthy controls (HC) showed that genes such as GFAP and ZEB2 were highly expressed in PD (Fig. 3F). The mean expression level of GFAP was 1.43 in healthy controls and 5.81 in PD patients, while the mean expression of ZEB2 was 3.18 in healthy controls and 3.86 in PD patients. Notably, the mean expression level of SLC18A2 in the SLC18A2+ subpopulation was 6.22, which was significantly higher than that in other subpopulations, all of which showed values no higher than 1.5. Further enrichment analysis suggests significant activation of pathways involving ubiquitin binding, dendritic spine function, and neuron migration in excitatory neurons (SLC18A2+) derived from Parkinson's disease (PD) patients compared to healthy control (HC) samples. In summary, we have identified a subpopulation of excitatory neurons (SLC18A2+) associated with PD, which may contribute to immune-related processes potentially linked to PD pathogenesis. The abnormality in dendritic spine-related pathways may be associated with synaptic loss and dysfunction in neural networks. Additionally, significant alterations in macro-autophagy and the regulation of autophagy suggest a decline in the cellular capacity to clear pathological protein aggregates.

4 Summary of Inhibitory Neuronal Subpopulations in Parkinson's Disease

**Fig. 3:**
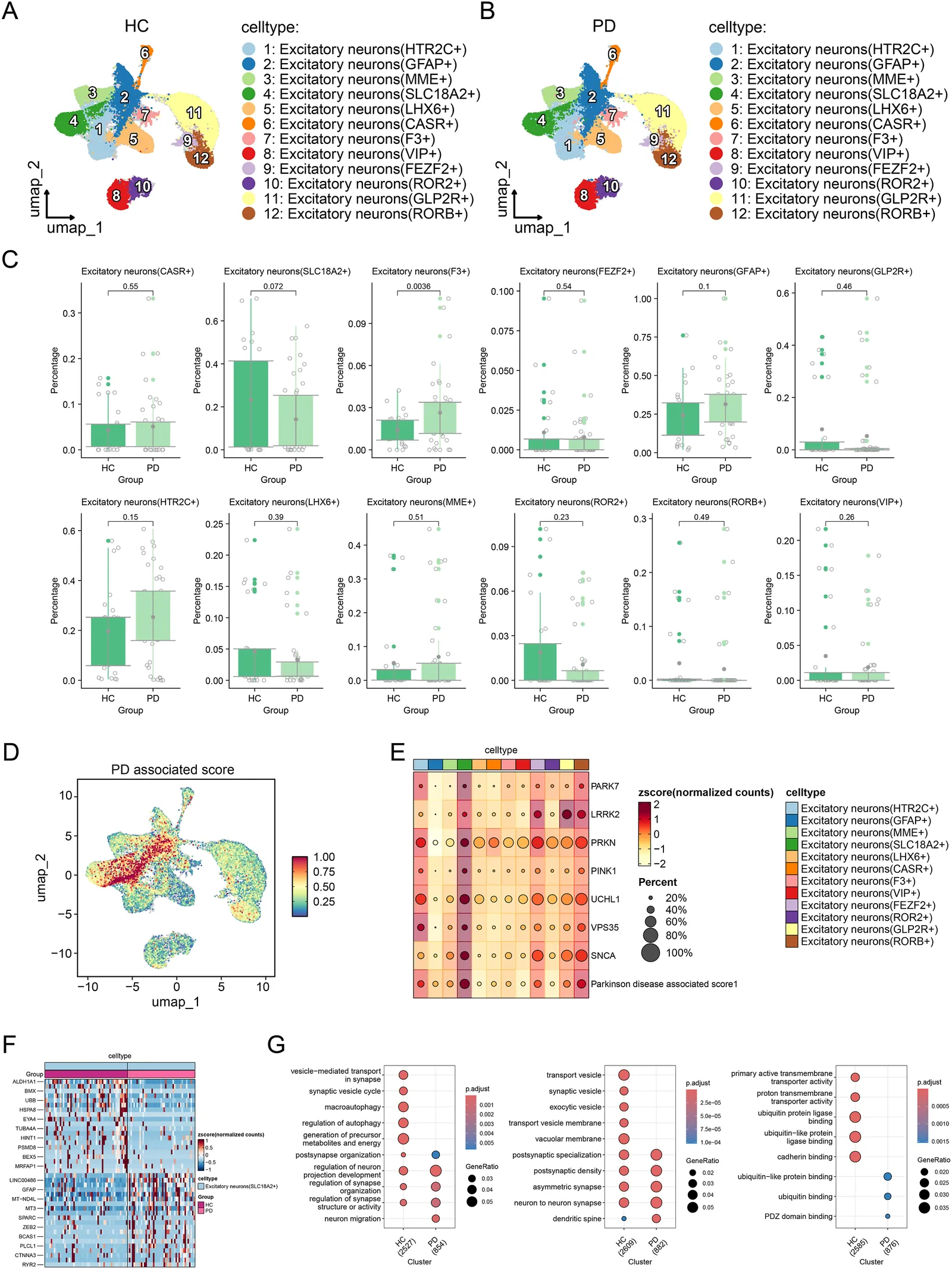
Alterations in Excitatory Neuron Subpopulations in PD Brains. (A–B) UMAP visualizations of excitatory neuron subpopulations in HC (A) and PD (B) samples, colored by cell type (defined by marker genes). (C) Box plots comparing the percentage of each excitatory neuron subpopulation between PD and HC, with P values (raw p value, *t*-test) indicating significant changes. (D) UMAP of excitatory neurons colored by a PD-associated score, highlighting subpopulations with high PD relevance (E) Dot plot of PD-associated genes (e.g., PARK7, LRRK2). across excitatory neuron subpopulations, showing normalized expression (color) and cell-type-specific percentage (dot size). (F) Heatmap of differentially expressed genes in excitatory neurons (SLC18A2+) between PD and HC, with normalized expression (color) and pathway enrichment (G) for biological processes, cell component and molecular function.

After subclustering inhibitory neurons, 10 related cell subpopulations were defined (Fig. 4A and B). Identification of PD-associated cell subpopulations based on PD-related genes revealed that the inhibitory neurons (SLC18A2+) subpopulation was associated with Parkinson's disease (PD) (Fig. 4C and D). The mean expression level of SLC18A2 in this subpopulation was 1.90, whereas the mean expression in all other subpopulations was no higher than 0.2. Pseudotime trajectory analysis of inhibitory neuronal subpopulations showed that inhibitory neurons (SLC18A2+) belong to an early stage of cellular development (Fig. 4E). Proportion analysis of cell subtypes suggests that the proportions of inhibitory neurons (EYA4+) and inhibitory neurons (HPSE2+) subpopulations were higher in PD patients compared to normal control samples. In contrast, the proportion of the inhibitory neurons (SLC18A2+) subpopulation was lower in PD patients than in normal samples (Fig. 4F). Compared with healthy controls (HC), Parkinson's disease (PD)-derived inhibitory neurons (SLC18A2+) cells exhibit markedly enhanced myelination/myelin-sheath and β-catenin-related processes, coupled with significant deficits in synaptic organization (Fig. 4G). In conclusion, the study identified specific inhibitory neuronal subpopulations that are associated with PD, with varying proportions and developmental stages. These insights contribute to a more nuanced understanding of the cellular landscape in PD and may support exploration of future therapeutic strategies targeting these specific subpopulations.

5 Oligodendrocytes (GAD1+) and Oligodendrocytes (SLC18A2+) were identified closely associated with PD

**Fig. 4:**
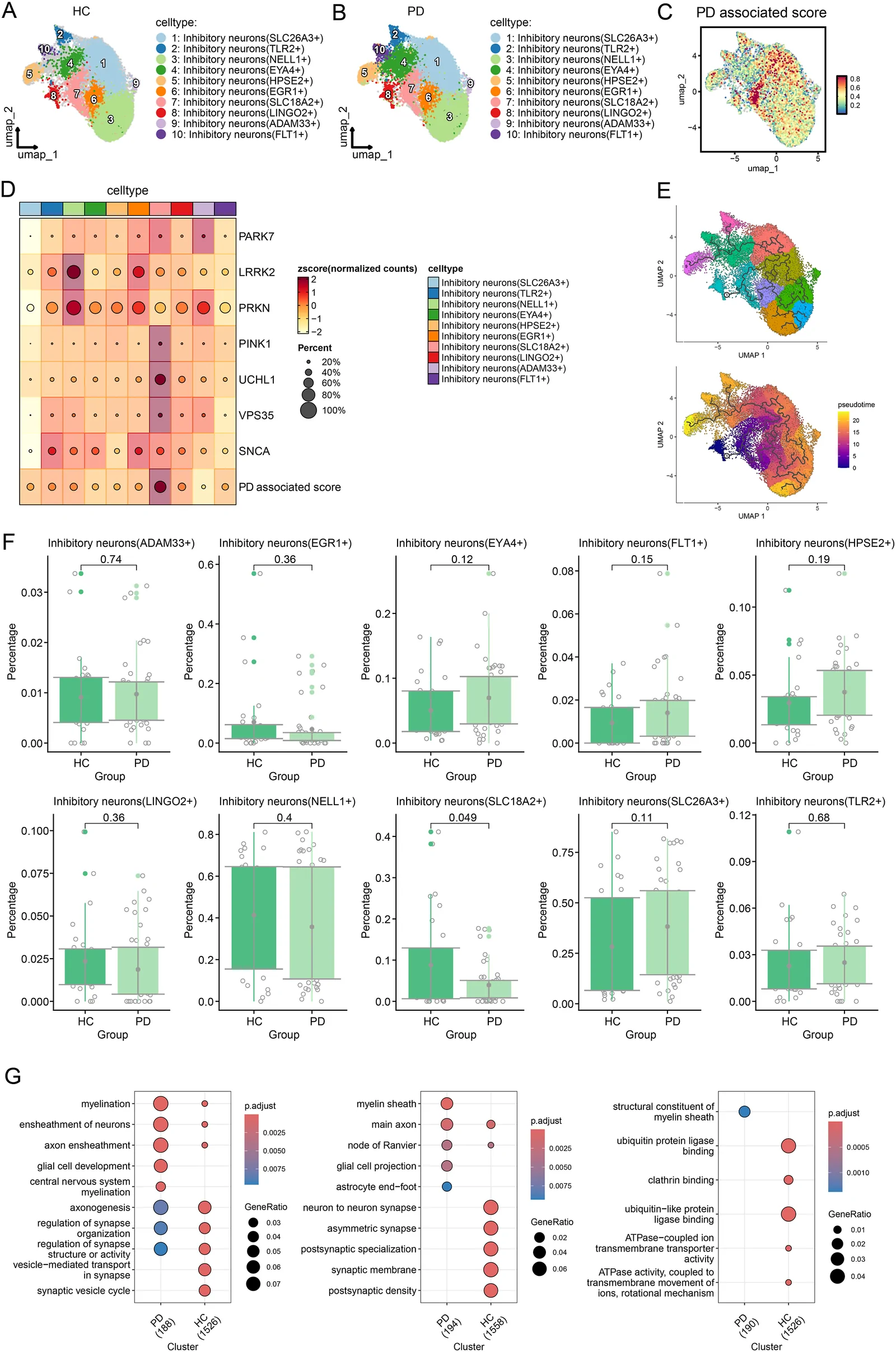
Inhibitory Neuron Subpopulations Show Pathway Dysregulation in PD. (A–B) UMAP visualizations of inhibitory neuron subpopulations in HC (A) and PD (B) samples, colored by cell type (defined by marker genes: SLC26A3+, TLR2+, etc.). (C) UMAP of inhibitory neurons colored by a PD-associated score, highlighting subpopulations with high PD relevance. (D) Dot plot of PD-associated genes across inhibitory neuron subpopulations, showing normalized expression (color) and cell-type-specific percentage (dot size). (E) UMAPs of inhibitory neurons colored by pseudotime (bottom) and cell clusters (top), illustrating developmental trajectories. (F) Box plots comparing the percentage of each inhibitory neuron subpopulation between PD and HC, with P values (Raw p value, *t*-test) indicating significant changes. (G) Bubble plots of enriched pathways in PD-associated inhibitory neurons, grouped by different origin (PD and HC), showing pathway enrichment (y-axis), -log10(adjusted P value), and gene ratio (dot size).

Oligodendrocytes were subclustered into 13 distinct cell subpopulations (Fig. 5A and B). Among these, the subpopulations most highly associated with Parkinson's disease (PD) were identified as Oligodendrocytes (GAD1+) and Oligodendrocytes (SLC18A2+) (Fig. 5C). PD-related marker genes, as shown in Fig. 5D, revealed that PINK1, VPS35, and UCHL1 were significantly highly expressed in these two oligodendrocyte subpopulations. Differential enrichment analysis of the Oligodendrocytes (GAD1+) subpopulation suggests that GAD1^+^ oligodendrocytes in PD exhibit a unique functional profile centered on dendrite morphogenesis, adherens junction, JUN kinase binding, GTPase regulator activity and nucleoside−triphosphatase regulator activity, may be linked to synaptic loss and circuit dysfunction (Fig. 5E). SLC18A2^+^ oligodendrocytes in PD display a functional program dominated by dendritic spine, miRNA binding and beta−catenin binding, implicating these cells in synaptic and axonal degeneration pathways (Fig. 5F). Additionally, the numbers of these two PD-associated oligodendrocyte subpopulations were significantly lower in PD patients than in normal samples (Fig. 5G), suggesting potential impairment or loss of related functions in PD patients.

6 SLC18A2^+^ astrocytes is a developmental and myelin-focused subpopulation depleted in parkinson's disease

**Fig. 5:**
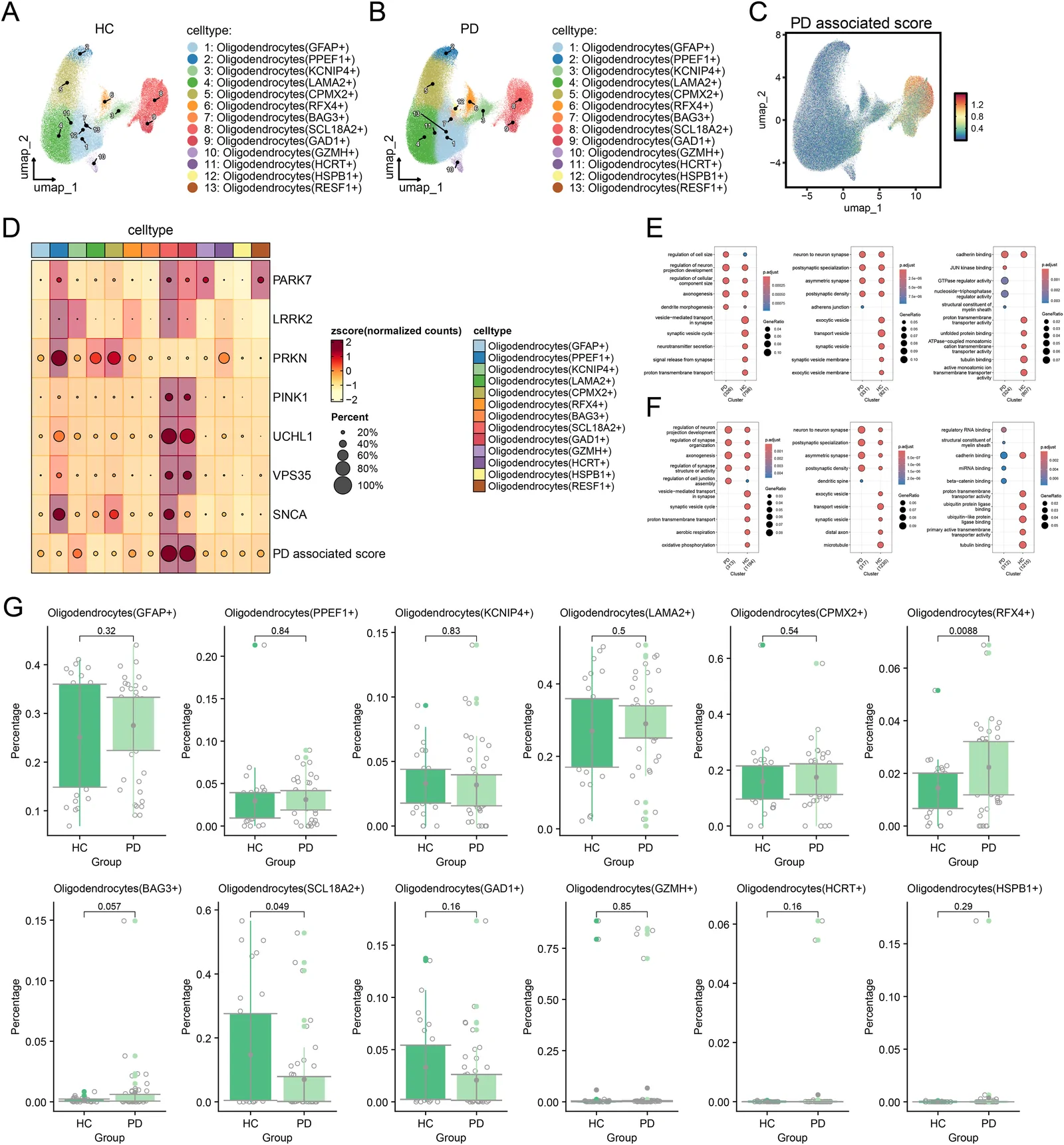
Oligodendrocyte Subpopulations in PD Show Altered PD-Associated Gene Expression. (A–B) UMAP visualizations of oligodendrocyte subpopulations in HC (A) and PD (B) samples, colored by cell type. (C) UMAP of oligodendrocytes colored by a PD-associated score, highlighting subpopulations with high PD relevance. (D) Dot plot of PD-associated genes' expression level across oligodendrocyte subpopulations, showing normalized expression (color) and cell-type-specific percentage (dot size). (E) Dot plots of enriched pathways in PD-associated oligodendrocytes (GAD1+). (F) Dot plots of enriched pathways in PD-associated oligodendrocytes (SLC18A2+). (G) Box plots comparing the percentage of each oligodendrocyte subpopulation between PD and HC, with P values (raw p value, *t*-test) indicating significant changes.

Sub clustering of all Astrocytes identified 10 distinct cell subpopulations (Fig. 6A and B). The expression levels of key Parkinson's disease - related genes (PARK7, LRRK2, etc.) showed distinct patterns in different astrocyte subpopulations (Fig. 6C&D). The astrocyte subpopulation with high expression of PD-associated genes was Astrocytes (SLC18A2+) (Fig. 6C and D). Enrichment analysis of this subpopulation suggests significant functional differences between PD patients and healthy controls. In PD patients, myelination, cell–cell contact zone, myelin sheath, β-catenin binding, and structural constituent of myelin sheath were markedly up-regulated (Fig. 6E). Consistent with previous reports, the PD-associated astrocyte subset SLC18A2+ was reduced in PD patients (Fig. 6F). Pseudotime analysis further suggests that SLC18A2+ astrocytes occupy an intermediate developmental state with elevated activity. Key genes including APBB1IP, ARHGAP15, DOCK8, and ELMO1 were highly expressed during early-to-mid development, whereas ADGRV1 expression peaked at later stages (Fig. 6G). These findings collectively suggest that the Astrocytes (SLC18A2+) subpopulation is associated with Parkinson's disease, with altered biological functions and gene expression patterns compared to normal states.

7 NK_CLIC3 centered immune–neuronal crosstalk and regional immune infiltration in Parkinson's disease

**Fig. 6:**
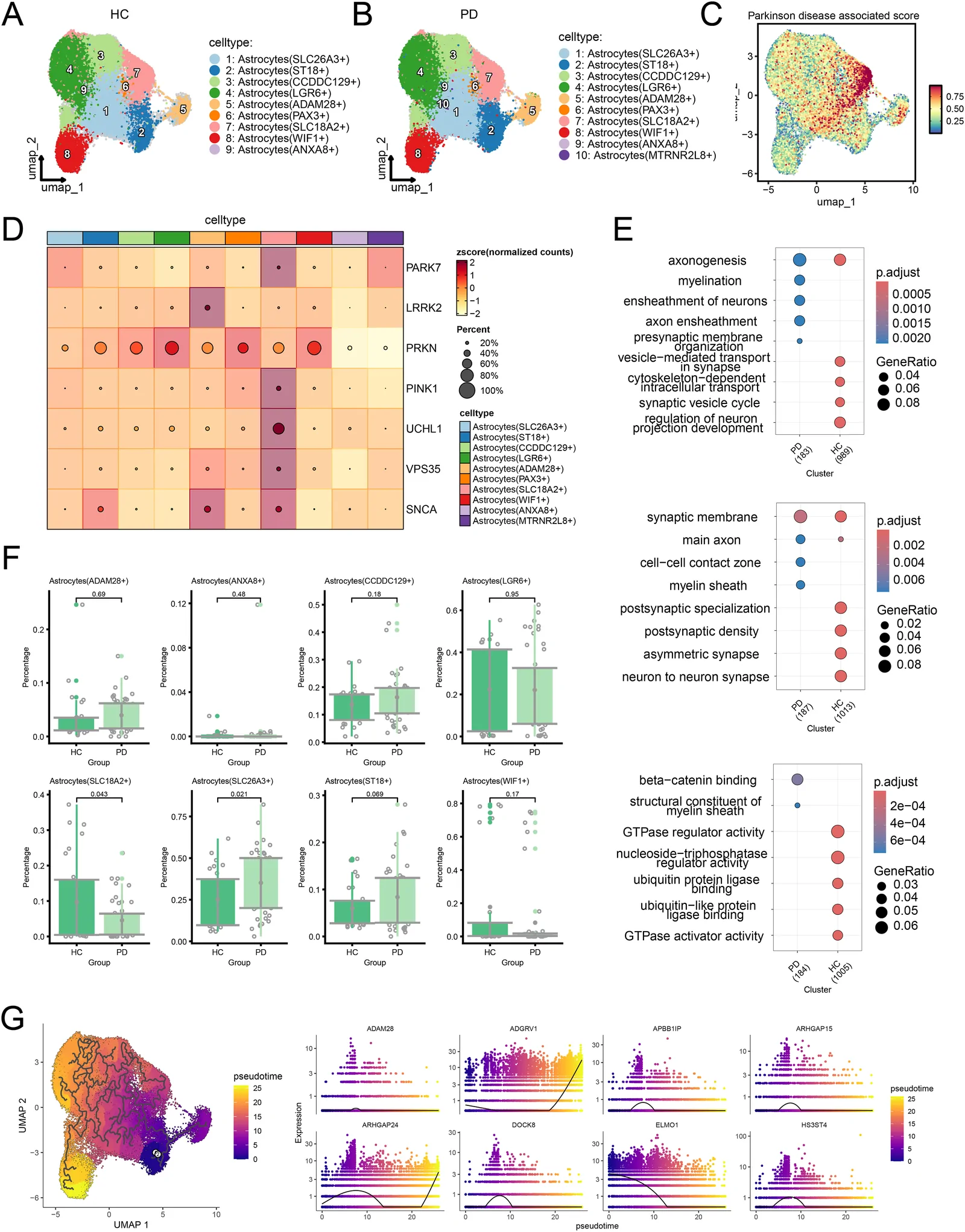
Astrocyte Subpopulations in PD Exhibit Dysregulated Pathways. (A–B) UMAP visualizations of astrocyte subpopulations in HC (A) and PD (B) samples, colored by cell type. (C) UMAP of astrocytes colored by a PD-associated score, highlighting subpopulations with high PD relevance. (D) Dot plot of PD-associated genes across astrocyte subpopulations, showing normalized expression (color) and cell-type-specific percentage (dot size). (E) Bubble plots of enriched pathways in PD-associated astrocytes, grouped by different origin (PD and HC), showing pathway enrichment (x-axis), -log10(adjusted P value), and gene ratio (dot size). (F) Box plots comparing the percentage of each astrocyte subpopulation between PD and HC, with P values (raw p value, *t*-test) indicating significant changes. (G) UMAP of astrocytes colored by pseudotime (left panel) and illustration of gene expression along pseudotime trajectories (right panel), illustrating dynamic changes in astrocytes.

NK_CLIC3 cells and all PD-associated cells, including excitatory neurons (SLC18A2^+^), inhibitory neurons (SLC18A2^+^), oligodendrocytes (SLC18A2^+^), and astrocytes (SLC18A2^+^), were extracted from PD samples for further analysis. To investigate the immune cell infiltration landscape in PD brain tissues, we examined cytokine expression and associated signaling pathways. Firstly, multiple cellular interactions were identified between these cell types, with the strongest interaction strength was observed between NK_CLIC3 and excitatory neurons (SLC18A2^+^) (Fig. 7A). Specifically, NK_CLIC3 cells exhibited extensive crosstalk with excitatory neurons, involving a total of 18 signaling pathways, while oligodendrocytes exhibited the fewest interactions with only approximately 7 pathways. Furthermore, NK_CLIC3 cells expressed IFN-γ, TNF-α, and TGF-β, with TGF-β being up-regulated (Fig. 7B). We further identified that oligodendrocytes (SLC18A2^+^) engage NK_CLIC3 cells via the THY1–ADGRE5 ligand–receptor axis, whereas the GZMA–PARD3 pathway may mediate NK_CLIC3-dependent repression of PARD3 expression (Fig. 7C). Immunohistochemistry (IHC) validated the expression of THY1, PARD3, and SLC18A2 in human brain samples (Fig. 7D–F). Extended immune-infiltration analysis revealed CD4^+^ T-cell, NK-cell, and M2 macrophage may infiltration in brain tissue (Fig. 7G). In Parkinson's disease, M2 macrophage infiltration was reduced in the amygdala and substantia nigra, whereas no difference was observed in the medial temporal gyrus relative to controls (Fig. 7H). NK_CLIC3 cells orchestrate a THY1/ADGRE5-GZMA/PARD3 immune–neuronal axis and TGF-β secretion which might represent a promising therapeutic strategy for restraining neuroinflammation.

8 The CLIC3-positive cell subset is capable of degrading α-syn.

**Fig. 7:**
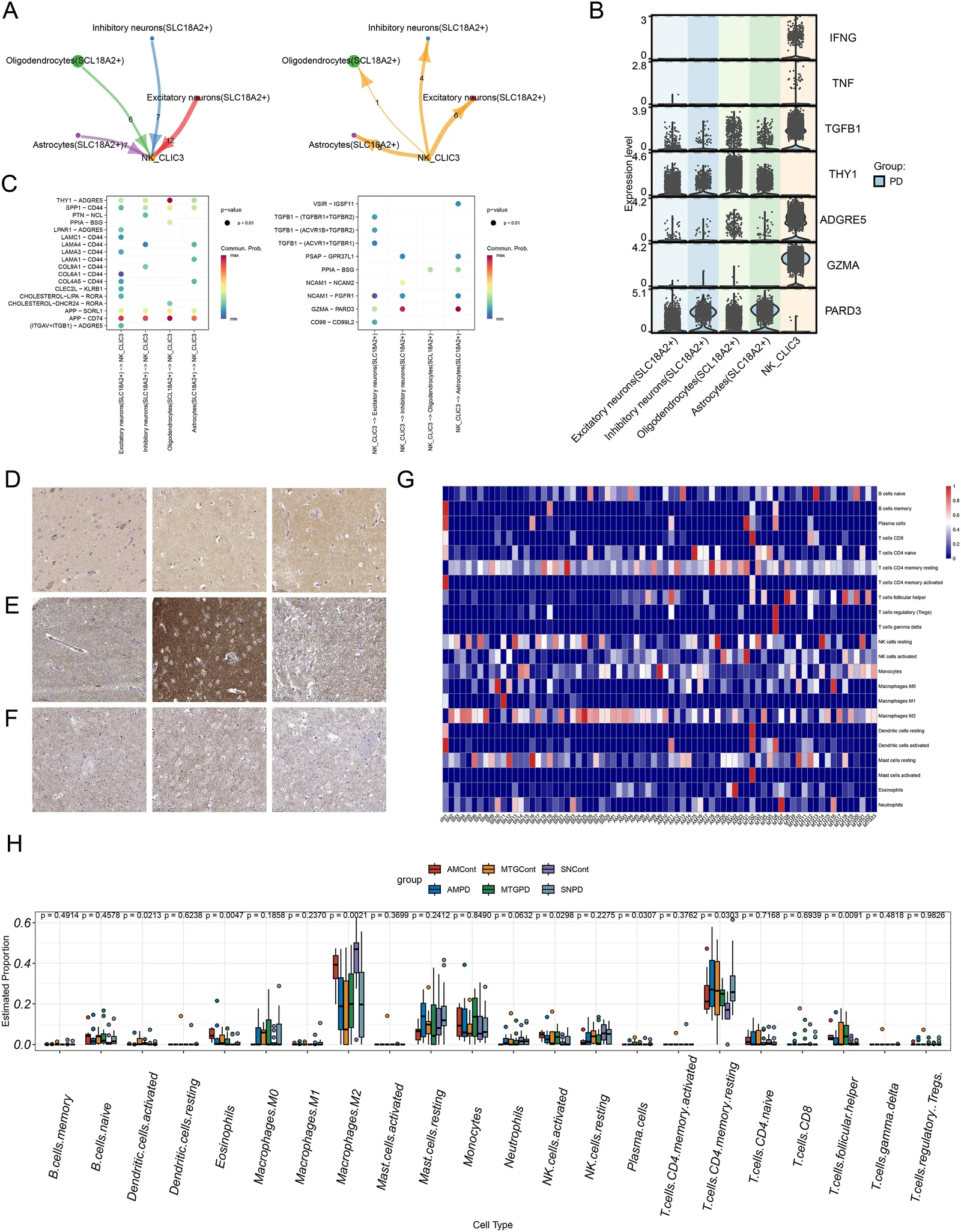
Cell-Cell Communication Analysis Reveals NK_CLIC3 Interactions with Neuronal Cells in PD. (A) Chord plots illustrate cell-cell communication networks involving NK_CLIC3 cells (center) and other cell types (SLC18A2+ inhibitory neurons, oligodendrocytes, astrocytes), with edges colored by interaction direction (source → target). (B) Violin plots of genes (IFNG, TNF and TGFB1 etc.) in NK_CLIC3 and interacting cell types, showing expression level in PD. (C) Dot plots of ligand-receptor pairs between NK_CLIC3 and target cells (all cells drive from PD), with interaction strength (color) and raw P values (dot size). (D–F) Immunohistochemistry (IHC) staining of brain tissue sections for key genes (D: THY1; E: PARD3; F: SLC18A2) of neuronal cells, highlighting protein level expression (scale bar: 50 μm). (G) Heatmap of Immune cell infiltration in different brain samples. (H) Bar plots of immune cell-type infiltration level in PD and HC brain samples (p value; Kruskal–Wallis H test).

NK cells (from a healthy donor) were isolated from the peripheral blood of a healthy donor via flow cytometry. A CD3-positive proportion of 3.13% indicated effective T-cell depletion (Fig. 8A), a CD56-positive proportion of 99.7% confirming the NK cell identity (Fig. 8B), and a CLIC3-positive proportion of 53.28% (Fig. 8C). Purified CLIC3+ NK cells were subsequently harvested and cultured for further experiments. To assess whether NK cells internalize extracellular α-syn, CLIC3+ NK cells were exposed to α-syn aggregates, and the internalization of insoluble α-syn species was analyzed by Western blotting. No detectable endogenous α-syn was observed in NK cells. Our in-vitro assays showed that CLIC3+ NK cells from a healthy donor internalized and degraded α-syn in vitro (Fig. 8D). To monitor the degradation of intracellular α-syn, CLIC3+ NK cells were incubated with extracellular α-syn species for 1 h, washed three times, and cultured for additional periods. Internalized α-syn species appeared to be degraded starting at 1 h post-incubation, diminished by 4 h, and nearly undetectable at 24 h. These findings indicate that healthy donor-derived CLIC3+ NK cells can clear α-syn protein in vitro.

**Fig. 8:**
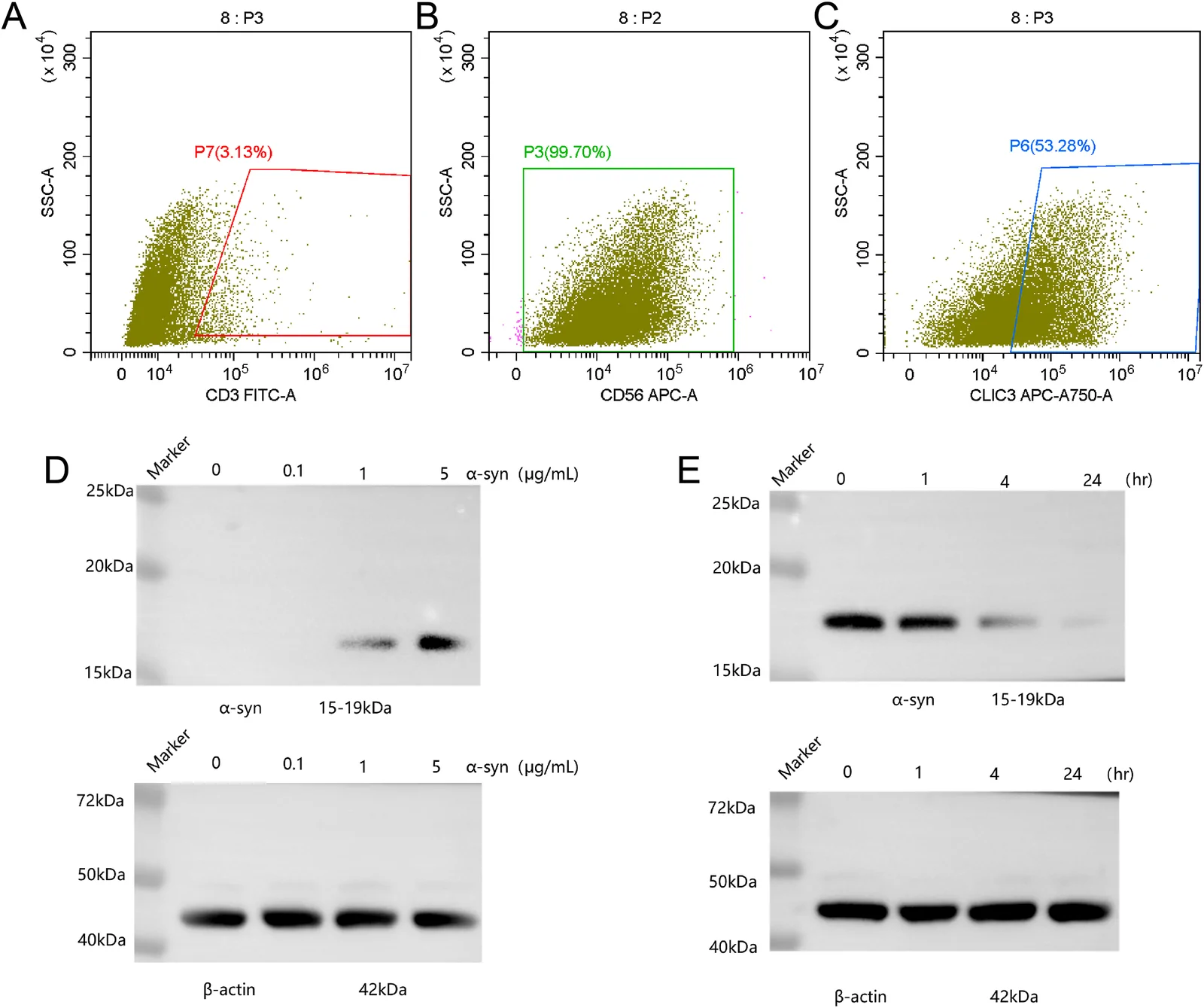
CLIC3+ NK cells from a healthy donor show in vitro α-syn clearance capacity. Healthy donor-derived NK cells were sorted to obtain CD3^−^CD56^+^CLIC3^+^ NK cells, with 3.13% CD3^+^ cells (A), 99.70% CD56^+^ cells (B), and 53.28% CLIC3^+^ cells (C); cells marked by colored boxes represent antibody-positive cells, and each dot indicates one individual cell. (D), Western blot analysis of internalized α-syn in CLIC3^+^ NK cells after incubation with the indicated concentrations of α-syn for 1 h. (E) Western blot analysis of α-syn degradation in CLIC3^+^ NK cells, which were treated with α-syn aggregates for 1 h, washed three times with fresh medium, and then cultured in fresh medium for up to 24 h.

## 4 Discussion

Parkinson's disease (PD), the second most prevalent neurodegenerative disease globally, has a complex pathological mechanism and lacks curative treatments [24]. Deep brain stimulation (DBS) represents an important therapeutic option for patients with advanced PD [25]. However, the biological underpinnings of its efficacy particularly its immunomodulatory effects have long remained incompletely elucidated. Through integrated multi-omics analysis, our study provides evidence suggesting that DBS may dampen neuroinflammation through immune-neuronal crosstalk potentially driven by the expansion of CLIC3^+^ natural killer (NK) cells, providing a novel perspective for understanding the mechanism of DBS efficacy.

Circulating lymphocytes have been shown to progressively decline in PD and can predict future motor symptom progression [26]. DBS, used as a symptomatic treatment, appears to counteract this progressive lymphocyte loss [27]. DBS drives a shift from pro-inflammatory CD4^+^ T helper 17 cells, which promote neurodegeneration, to anti-inflammatory CD4^+^ regulatory T cells, thereby mitigating neuroinflammation [27]. These findings suggest that DBS can modulate both central and peripheral components of the immune system, leading to clinical improvements through immunosuppressive/anti-inflammatory mechanisms [28], [29]. In this study, we identified that DBS significantly upregulates CLIC3 expression. PD patients exhibit reduced M2-type macrophage infiltration, resulting in impaired anti-inflammatory capacity. DBS-associated elevation of NK_CLIC3 may compensate for this deficit by enhancing anti-inflammatory functions, thereby alleviating neuroinflammation in PD patients and may confer therapeutic benefits. Previous studies have demonstrated that NK cells contribute to inhibiting the progression of Parkinson's disease [30]. Extending these findings, our data further suggest that the therapeutic effects of DBS might be partly mediated by CLIC3^+^ NK-cell-dependent clearance of α-synuclein.

Transcriptomic analyses of brain cells strongly support the existence of brain-resident NK cells, identifying NK cells as a small but distinct and biologically significant cell cluster within the brain parenchyma [31], [32], [33]. NK cells may play a role in clearing α-synuclein aggregates—the primary component of Lewy bodies that contribute to neuronal degeneration in PD [34]. A recent study demonstrated neuroprotective effects of NK cells in PD [30], showing that NK cells reduce α-synuclein burden in vitro, and systemic depletion of NK cells in a preclinical mouse model of PD results in increased pathological α-synuclein accumulation in multiple brain regions, including the striatum, substantia nigra pars compacta (SNpc), and brainstem [30]. Several studies have implicated interferon-γ (IFN-γ) levels in PD, with recent evidence showing elevated plasma IFN-γ levels in PD patients [35]. NK cells are major producers of IFN-γ [36]. In the central nervous system (CNS), IFN-γ improves symptoms of autoimmune diseases such as experimental autoimmune encephalomyelitis (EAE), suggesting potential neuroprotective effects [37], [38]. With age-related reductions in IFN-γ production by NK cells, there may be detrimental impacts on the ability of antigen-presenting cells (APCs) to manage pathogens (α-synuclein aggregates) both centrally and peripherally [39], [40].

Vesicular monoamine transporter 2 (VMAT2), encoded by SLC18A2, is critical for packaging monoamine neurotransmitters into synaptic vesicles; its dysregulation is associated with schizophrenia, mood disorders, and PD [41]. Recently, SLC18A2 (VMAT2) was identified as a key hub gene among inflammation-related differentially expressed genes in PD [42], [43]. Additionally, SLC18A2 has been linked to infantile-onset parkinsonism-dystonia 2, representing a novel genotype [44], [45], [46]. In this study, PD-associated cell subpopulations exhibited high SLC18A2 expression. Functional imbalance in Parkinson's disease is characterized by a consistent up-regulation of myelination, myelin-sheath organization, β-catenin signaling, and ubiquitin-protein-ligase-mediated autophagy stress across all analyzed neural subpopulations, concomitant with a marked loss of synaptic-vesicle cycling, dendritic-spine maintenance and postsynaptic specialisation.

Synaptic transmission dysfunction often precedes the massive loss of dopaminergic neurons in early-stage PD. The Par3–Par6–aPKC complex plays a central role in maintaining presynaptic/postsynaptic polarity, AMPA/NMDA receptor trafficking, and synaptic plasticity. Abnormal expression or localization of PARD3 can lead to synaptic structural and functional disorders, potentially underlying early cognitive and motor circuit impairments in PD [47]. GZMA in the lymphocytes could downregulate the expression of PARD3 through direct interaction with PARD3 [48]. We found that NK_CLIC3 cells down-regulate PARD3 in SLC18A2^+^ neuronal subsets via the GZMA–PARD3 axis, whereas these same neurons engage NK_CLIC3 through the THY1(CD90)–ADGRE5(CD97) ligand–receptor pair. Although the CD90–CD97 axis has been shown to drive MAPK/ERK-glycolytic programs in glioblastoma [49] and JAK2–STAT5-mediated stem-cell survival in myeloproliferative neoplasms [50], its precise functional contribution to Parkinson's disease remains to be elucidated.

This study has several limitations. Importantly, the in-vitro experiments assessing α-synuclein degradation by CLIC3^+^ NK cells used blood from only one healthy donor, rather than NK cells isolated from Parkinson's disease patients. As such, these results cannot directly demonstrate that patient-derived CLIC3^+^ NK cells exert identical α-synuclein-clearing effects under disease-relevant pathological conditions. PD-associated inflammatory milieu may remodel NK-cell functionality. Further studies using CLIC3^+^ NK cells purified from multiple PD patients are needed to validate this function, and our conclusions regarding α-synuclein clearance by CLIC3^+^ NK cells should be interpreted cautiously. Also, the specific molecular mechanisms by which CLIC3 regulates NK cell function and the in vitro validation of the THY1-ADGRE5 and GZMA-PARD3 pathways remain incomplete and require further investigation using cell co-culture and gene knockout experiments. Finally, the dose-effect relationship between DBS parameters (e.g., stimulation frequency, intensity) and NK_CLIC3 expansion remains unclear and necessitates refined analysis.

In summary, our findings suggest that CLIC3+ NK cells may have α-syn-clearing potential, but this requires validation in PD patient-derived cells and in disease-relevant models. Whether DBS promotes this function in patient-derived CLIC3+ NK cells remains to be determined. Future research may focus on in vitro expansion techniques for NK_CLIC3 and the development of CLIC3-targeted drugs, advancing PD treatment from symptomatic intervention toward disease-modifying therapy.

## Authors' contributions

Z.S. (Zhang Shushan), L.Z. (Luo Zhanghong), and Y.T. (Yishuai Tan) designed the study, performed data analysis (including microarray data, bulk RNA-seq, single-cell RNA-seq, and Mendelian randomization analyses), and drafted the initial manuscript. C.B. (Cheng Bo) and X.W. (Xiao Wei) contributed to data collection, quality control of sequencing datasets, and visualization of results (preparing Fig. 1, Fig. 2, Fig. 3, Fig. 4). J.G. (Jiang Guohui) assisted with cell-cell communication analysis and validation of ligand-receptor interactions (supporting Fig. 7). Z.T. (Zhu Tao) conceived the project, supervised the analysis, revised the manuscript critically for intellectual content, and secured funding. All authors reviewed and approved the final version of the manuscript.

## Ethics declaration

Written informed consent to take part in the study and to publish the article has been obtained from all participants or their legal representatives. The privacy rights of participants have been observed.

This study included organ or tissue donors. This study includes human biological material and consent was obtained by donors, or their next of kin or legal representatives, for use in this study and for publication of the article. The samples used in this research were not sourced from executed prisoners or prisoners of conscience.

This study was performed in compliance with relevant laws, regulatory frameworks and guidelines where the research took place. Ethics committee approval was not required under relevant laws and institutional guidelines. One blood sample from a healthy volunteer was collected with verbal informed consent. This low-risk sampling was exempt from requirements for written consent and formal ethical review per relevant local institutional rules.

## Consent for publication

The work described in this manuscript is entirely original and has not been published previously, except maybe in the form of an abstract or as part of a published lecture, review, or thesis.

## Code availability

All custom analysis codes are publicly available at https://github.com/RDGank/Parkinson.

## Funding

Nanchong 14th Five-Year Plan for Social Science Research Project(NC25B186); the Key Project of the Primary 10.13039/100018696Health Development Research Center of Sichuan Province Program(SWFZ23-Z-10); the 10.13039/501100018542Natural Science Foundation of Sichuan Province (2024NSFSC0626); the 10.13039/100006190Research and Development Project of Affiliated Hospital of North Sichuan Medical College(2022MPZK0010).

## Declaration of competing interest

The authors declare that they have no competing interests or personal relationships that could have appeared to influence the work reported in this paper.

## Data Availability

Microarray gene expression profiling data used in this study was obtained from the Gene Expression Omnibus (GEO) database with the accession number GSE23676. Single-cell/single-nucleus RNA-seq (scRNA/snRNA-seq) datasets were also downloaded from the GEO database, with accession numbers GSE184950, GSE202210, GSE223138, and GSE243639. The gene expression dataset for immune cell infiltration analysis was GSE114517 from the GEO database, which included bulk RNA-seq data of 75 brain tissue samples. Summary-level data for exposure and outcome traits were retrieved from public genome-wide association study (GWAS) repositories (IEU OpenGWAS). Specifically, the expression quantitative trait locus (eQTL) data for CLIC3 (exposure; ID: eqtl-a-ENSG00000169583) included 31,684 samples and 18,471 single nucleotide polymorphisms (SNPs). Three independent GWAS datasets related to Parkinson's disease (PD, outcome) were used: ieu-a-812 (n = 5,691; 453,218 SNPs), finn-b-PD_DEMENTIA (n = 216,895; 16,380,459 SNPs), and finn-b-PD_DEMENTIA_EXMORE (n = 111,888; 16,379,396 SNPs).

## References

[1] Kefalakes E., Mewes F., Peristich D., Plötner C., Shcherbatyy V., Schipke J., Schneider F., Käufer C., Rumpel R. (2025). A holistic rat model to investigate therapeutic interventions in parkinson's disease: viral induction of a slow-progressing motor phenotype, dopaminergic degeneration and early microglia neuroinflammation. Brain Res. Bull..

[2] Vaquero-Rodríguez A., Razquin J., Murueta-Goyena A., Miguelez C., Ruíz-Ortega J., Lafuente J.V., Bengoetxea H., Ortuzar N. (2025). Temporal progression of pathological features in an α-synuclein overexpression model of parkinson's disease. Brain Struct. Funct..

[3] Angelopoulou E., Paudel Y.N., Bougea A., Piperi C. (2021). Impact of the apelin/APJ axis in the pathogenesis of parkinson's disease with therapeutic potential. J. Neurosci. Res..

[4] Regensburger M., Csoti I., Jost W.H., Kohl Z., Lorenzl S., Pedrosa D.J., Lingor P. (2026). Motor and non-motor fluctuations in parkinson's disease: the knowns and unknowns of current therapeutic approaches. J. Neural Transm..

[5] Xie S., Liu Y., Yang A., Meng F., Jiang C., Fang H., Han R., Zhang J., Shi L. (2025). Scalp block improves electrophysiological stability and patient cooperation during deep brain stimulation surgery. Sci. Rep..

[6] Krauss J.K., Lipsman N., Aziz T., Boutet A., Brown P., Chang J.W., Davidson B., Grill W.M., Hariz M.I., Horn A. (2021). Technology of deep brain stimulation: current status and future directions. Nat. Rev. Neurol..

[7] Muller M., van Leeuwen M.F.C., Hoffmann C.F., van der Gaag N.A., Zutt R., van der Gaag S., Schouten A.C., Contarino M.F. (2025). From subthalamic local field potentials to the selection of chronic deep brain stimulation contacts in parkinson's disease - a systematic review. Brain Stimul..

[8] Sun Y., Wei K., Liao X., Wang J., Gao L., Pang B. (2025). Lipid metabolism in microglia: emerging mechanisms and therapeutic opportunities for neurodegenerative diseases. Int. J. Mol. Med..

[9] Little J., Barakat-Haddad C., Martino R., Pringsheim T., Tremlett H., McKay K.A., van Lieshout P., Walsh S.J., Gomes J., Krewski D. (2017). Genetic variation associated with the occurrence and progression of neurological disorders. Neurotoxicology.

[10] Xie Y., Chen L., Chen J., Chen Y. (2024). Calcitriol restrains microglial M1 polarization and alleviates dopaminergic degeneration in hemiparkinsonian mice by boosting regulatory T-cell expansion. Brain Behav.

[11] Kim H.W., Wang S., Davies A.J., Oh S.B. (2023). The therapeutic potential of natural killer cells in neuropathic pain. Trends Neurosci..

[12] Zhu X., Wang C.L., Yu J.F., Weng J., Han B., Liu Y., Tang X., Pan B. (2023). Identification of immune-related biomarkers in peripheral blood of schizophrenia using bioinformatic methods and machine learning algorithms. Front. Cell. Neurosci..

[13] Wang Q., Wang M., Choi I., Sarrafha L., Liang M., Ho L., Farrell K., Beaumont K.G., Sebra R., De Sanctis C. (2024). Molecular profiling of human substantia nigra identifies diverse neuron types associated with vulnerability in parkinson's disease. Sci. Adv..

[14] Zhu B., Park J.M., Coffey S.R., Russo A., Hsu I.U., Wang J., Su C., Chang R., Lam T.T., Gopal P.P. (2024). Single-cell transcriptomic and proteomic analysis of parkinson's disease brains. Sci. Transl. Med..

[15] Martirosyan A., Ansari R., Pestana F., Hebestreit K., Gasparyan H., Aleksanyan R., Hnatova S., Poovathingal S., Marneffe C., Thal D.R. (2024). Unravelling cell type-specific responses to parkinson's disease at single cell resolution. Mol. Neurodegener..

[16] Soreq L., Bergman H., Goll Y., Greenberg D.S., Israel Z., Soreq H. (2012). Deep brain stimulation induces rapidly reversible transcript changes in Parkinson's leucocytes. J. Cell Mol. Med..

[17] Xiong L.L., Du R.L., Niu R.Z., Xue L.L., Chen L., Huangfu L.R., Cai X.X., He X.Y., Huang J., Huang X.Y. (2024). Single-cell RNA sequencing reveals peripheral immunological features in parkinson's disease. npj Parkinson's Dis..

[18] Korsunsky I., Millard N., Fan J., Slowikowski K., Zhang F., Wei K., Baglaenko Y., Brenner M., Loh P.R., Raychaudhuri S. (2019). Fast, sensitive and accurate integration of single-cell data with harmony. Nat. Methods.

[19] Xu S., Hu E., Cai Y., Xie Z., Luo X., Zhan L., Tang W., Wang Q., Liu B., Wang R. (2024). Using clusterProfiler to characterize multiomics data. Nat. Protoc..

[20] Simón-Sánchez J., Schulte C., Bras J.M., Sharma M., Gibbs J.R., Berg D., Paisan-Ruiz C., Lichtner P., Scholz S.W., Hernandez D.G. (2009). Genome-wide association study reveals genetic risk underlying parkinson's disease. Nat. Genet..

[21] Cao J., Spielmann M., Qiu X., Huang X., Ibrahim D.M., Hill A.J., Zhang F., Mundlos S., Christiansen L., Steemers F.J. (2019). The single-cell transcriptional landscape of Mammalian organogenesis. Nature.

[22] Jin S., Plikus M.V., Nie Q. (2025). CellChat for systematic analysis of cell-cell communication from single-cell transcriptomics. Nat. Protoc..

[23] Simchovitz A., Hanan M., Yayon N., Lee S., Bennett E.R., Greenberg D.S., Kadener S., Soreq H. (2020). A lncRNA survey finds increases in neuroprotective LINC-PINT in parkinson's disease substantia nigra. Aging Cell.

[24] Slouha E., Ibrahim F., Esposito S., Mursuli O., Rezazadah A., Clunes L.A., Kollias T.F. (2024). Botulinum toxin for the management of parkinson's disease: a systematic review. Cureus.

[25] Martínez-Fernández R., Paschen S., Del Álamo M., Rodríguez-Rojas R., Pineda-Pardo J.A., Blesa J., Kaplitt M.G., Deuschl G., Obeso J.A. (2025). Focused ultrasound therapy for movement disorders. Lancet Neurol..

[26] Kim R., Kang N., Byun K., Park K., Jun J.S. (2023). Prognostic significance of peripheral neutrophils and lymphocytes in early untreated parkinson's disease: an 8-year follow-up study. J. Neurol. Neurosurg. Psychiatry.

[27] McFleder R.L., Musacchio T., Keller J., Knorr S., Petschner T., Chen J.Z., Muthuraman M., Badr M., Harder-Rauschenberger L., Kremer F. (2025). Deep brain stimulation halts Parkinson's disease-related immune dysregulation in the brain and peripheral blood. Brain Behav. Immun..

[28] Asimakidou E., Sidiropoulos C. (2025). Immunomodulatory effects of invasive and non-invasive brain stimulation in parkinson's disease. Parkinsonism Relat. Disorders.

[29] Grembecka B., Majkutewicz I., Harackiewicz O., Wrona D. (2023). Deep-brain subthalamic nucleus stimulation enhances food-related motivation by influencing neuroinflammation and anxiety levels in a rat model of early-stage parkinson's disease. Int. J. Mol. Sci..

[30] Earls R.H., Menees K.B., Chung J., Gutekunst C.A., Lee H.J., Hazim M.G., Rada B., Wood L.B., Lee J.K. (2020). NK cells clear α-synuclein and the depletion of NK cells exacerbates synuclein pathology in a mouse model of α-synucleinopathy. Proc. Natl. Acad. Sci. U. S. A..

[31] Goleij P., Amini A., Tabari M.A.K., Hadipour M., Sanaye P.M., Alsharif K.F., Daglia M., Larsen D.S., Khan H. (2025). The role of interleukin (IL)-2 cytokine family in parkinson's disease. Cytokine.

[32] Li Q., Cheng Z., Zhou L., Darmanis S., Neff N.F., Okamoto J., Gulati G., Bennett M.L., Sun L.O., Clarke L.E. (2019). Developmental heterogeneity of microglia and brain myeloid cells revealed by deep single-cell RNA sequencing. Neuron.

[33] Van Hove H., Martens L., Scheyltjens I., De Vlaminck K., Pombo Antunes A.R., De Prijck S., Vandamme N., De Schepper S., Van Isterdael G., Scott C.L. (2019). A single-cell atlas of mouse brain macrophages reveals unique transcriptional identities shaped by ontogeny and tissue environment. Nat. Neurosci..

[34] Daneshjou D., Nabavi S.M., Shams P., Faranoush P., Salari M., Ebrahimi M. (2025). The double-edged sword role of natural killer cells in parkinson's disease. Cell. Immunol..

[35] Mount M.P., Lira A., Grimes D., Smith P.D., Faucher S., Slack R., Anisman H., Hayley S., Park D.S. (2007). Involvement of interferon-gamma in microglial-mediated loss of dopaminergic neurons. J. Neurosci..

[36] Paolini R., Bernardini G., Molfetta R., Santoni A. (2015). NK cells and interferons. Cytokine Growth Factor Rev..

[37] Voorthuis J.A., Uitdehaag B.M., De Groot C.J., Goede P.H., van der Meide P.H., Dijkstra C.D. (1990). Suppression of experimental allergic encephalomyelitis by intraventricular administration of interferon-gamma in lewis rats. Clin. Exp. Immunol..

[38] Ottum P.A., Arellano G., Reyes L.I., Iruretagoyena M., Naves R. (2015). Opposing roles of interferon-gamma on cells of the central nervous system in autoimmune neuroinflammation. Front. Immunol..

[39] Di Lorenzo G., Balistreri C.R., Candore G., Cigna D., Colombo A., Romano G.C., Colucci A.T., Gervasi F., Listì F., Potestio M. (1999). Granulocyte and natural killer activity in the elderly. Mech. Ageing Dev..

[40] Earls R.H., Lee J.K. (2020). The role of natural killer cells in parkinson's disease. Exp. Mol. Med..

[41] Wei F., Liu H., Zhang W., Wang J., Zhang Y. (2025). Drug inhibition and substrate transport mechanisms of human VMAT2. Nat. Commun..

[42] Shen Z., Zhang J., Jing X., Tao E. (2025). Construction and identification of inflammation-related TF-mRNA-miRNA coexpression network and immune infiltration in parkinson's disease. Parkinsons Dis..

[43] Gu L., Wang X., Liu Y., Tang H., Gu J., Wang W., Lu X., Lou M. (2026). Identification of hub genes and prediction of interacting chemicals in parkinson's disease using bioinformatics. Curr. Med. Chem..

[44] Janardhanan A., Mahale R., Mahima R., Goyal S., Chanda B., Udupi G., Pooja M., Pavagada M., Padmanabha H. (2025). SLC18A2 associated infantile-onset- parkinsonism-dystonia 2: a novel genotype. Parkinsonism Relat. Disorders.

[45] Rosenthal L.S., Farag M., Aziz N.A., Bang J. (2025). Vesicular monoamine transport inhibitors: current uses and future directions. Lancet.

[46] Manohar S., Jacobe J., Berger R., Jankovic J., Hull M. (2025). Real-world experiences with VMAT2 inhibitors in pediatric hyperkinetic movement disorders. Tremor Other Hyperkinet Mov (N Y).

[47] Zhang L., Wei X. (2022). The roles of Par3, Par6, and aPKC polarity proteins in normal neurodevelopment and in neurodegenerative and neuropsychiatric disorders. J. Neurosci..

[48] Jiang X., Chen N., Wei Q., Luo X., Liu X., Xie L., Yi P., Xu J. (2025). Single-cell RNA sequencing and cell-cell communication analysis reveal tumor microenvironment associated with chemotherapy responsiveness in ovarian cancer. Clin. Transl. Oncol..

[49] Ravn-Boess N., Roy N., Hattori T., Bready D., Donaldson H., Lawson C., Lapierre C., Korman A., Rodrick T., Liu E. (2023). The expression profile and tumorigenic mechanisms of CD97 (ADGRE5) in glioblastoma render it a targetable vulnerability. Cell Rep..

[50] Ivanov D., Milosevic Feenstra J.D., Sadovnik I., Herrmann H., Peter B., Willmann M., Greiner G., Slavnitsch K., Hadzijusufovic E., Rülicke T. (2023). Phenotypic characterization of disease-initiating stem cells in JAK2- or CALR-Mutated myeloproliferative neoplasms. Am. J. Hematol..

